# Targeting PERK-ATF4-P21 axis enhances the sensitivity of osteosarcoma HOS cells to Mppα-PDT

**DOI:** 10.18632/aging.205511

**Published:** 2024-02-05

**Authors:** Shenxi Zhong, Ye Zhang, Hai Mou, Changchun Jian, Qiu Huang, Yunsheng Ou

**Affiliations:** 1 Department of Orthopedics, The First Affiliated Hospital of Chongqing Medical University, Yuzhong, Chongqing 400016, China; 2 Orthopedic Laboratory of Chongqing Medical University, Yuzhong, Chongqing 400016, China

**Keywords:** MPPα-PDT, human osteosarcoma, PERK pathway, autophagy, apoptosis, p21

## Abstract

Osteosarcoma (OS) is the most prevalent type of malignant bone tumor in adolescents. The overall survival of OS patients has reached a plateau recently. Thus, there is an urgent need to develop approaches to improve the sensitivity of OS to therapies. Pyropheophorbide-α methyl ester-mediated photodynamic therapy (MPPα-PDT) is a new type of tumor therapy, and elucidating its mechanism is helpful to improve its anti-tumor efficacy. Here, we investigated how PERK signaling promotes the human OS (HOS) cell survival induced by MPPα-PDT, as overcoming this may enhance sensitivity to MPPα-PDT. We found that MPPα-PDT combined with PERK inhibitor GSK2656157 enhanced HOS cell apoptosis by suppressing autophagy and p21. Autophagy inhibition and p21 depletion enhanced cell death, indicating pro-survival effects in MPPα-PDT. Notably, p21 was found to be an effector of the PERK-Atf4 pathway, which could positively regulate autophagy mediated by MPPα-PDT. In conclusion, we found that the combination of MPPα-PDT and GSK2656157 enhanced apoptosis in HOS cells by inhibiting autophagy. Mechanistically, this autophagy is p21-dependent and can be suppressed by GSK2656157, thereby enhancing sensitivity to MPPα-PDT.

## INTRODUCTION

Osteosarcoma (OS), the most common bone malignancy in adolescents and young adults, is characterized by a high rate of lung metastases [1]. Despite advances in treatment with a combination of chemotherapy and surgery, its 5-year survival rate remains low [2, 3]. Thus, there is an urgent need for more effective OS therapies. Photodynamic therapy (PDT) is a novel anti-tumor approach that relies on photosensitization transferring excited-state energy to cellular oxygen during irradiation at a specific wavelength. Cytotoxic reactive oxygen species (ROS) are produced during this procedure [4, 5]. Pyropheophorbide-α methyl ester (MPPα) is a new type of second-generation photosensitizer that is characterized by its high stability and high concentration in tumors [6]. To date, MPPα-PDT has been shown to possess anti-tumor properties against OS, lung cancer, and breast cancer [7–9]. However, it may also trigger resistance to treatment. In our previous work, we have proved that MPPα-PDT triggers processes including pumping out of intracellular MPPα and ROS, contributing to adaption and even resistance to MPPα-PDT [10, 11].

The endoplasmic reticulum (ER), the production site for secreted or membrane-associated proteins, is a subcellular entity that dynamically reacts to stress signals to control overall protein synthesis. Various cellular challenges such as ROS, hypoxia, and nutrient deprivation may disrupt ER reduction/oxidation (redox) regulation as well as homeostasis. This may lead to ER stress and an increasing number of misfolded and unfolded proteins, also referred to as the unfolded protein response (UPR) [12]. Physiologically, ER stress is an adaptive response to physiological challenges that promotes cell survival. If the stressor is overly intense or persists for an extended period, the accumulation of unfolded or misfolded proteins in the ER triggers the relevant ER stress signaling pathway, leading to cell apoptosis [13]. The primary modulators and immediate enablers of the ER stress response mechanism include protein kinase RNA-like ER kinase (PERK), eukaryotic initiation factor 2α (eIF2α), activating transcription factor 4 (ATF4), and C/EBP homologous protein (CHOP). When ER stress occurs, GRP78 separates from these proteins and activates their downstream signaling pathways, respectively, to rebuild the functional stability of the ER and enhance the adaptability of cells to adverse stimuli. During this process, PERK forms a dimer and autophosphorylates, generating its active, phosphorylated form. Further phosphorylations modify eIF2α, which inhibits ER mRNA translation and protein synthesis functions. Stress sensors in the ER lumen detect ROS, which in turn intensify UPR signaling [14, 15]. By phosphorylating and stabilizing NRF2, PERK reduces ROS accumulation, which controls the expression of phase II detoxification enzymes induced by ROS, such as glutathione S-transferase and HO-1 [16]. Given that PDT targeting triggers cancer cell death via intracellular ROS generation, the PERK pathway presents a plausible candidate for a resistance mechanism.

Autophagy, a conserved and regulated catabolic process, eliminates cytosolic material such as damaged organelles and long-lived proteins [17]. In specific situations, it may serve as a vital factor contributing to pro-survival adaptive reactions. However, previous studies have presented conflicting evidence on the effects of autophagy on apoptosis, and the functions of autophagy in tumorigenesis and anti-cancer treatments remain paradoxical. Autophagy has been reported to inhibit cell apoptosis and promote tumor survival. However, other studies indicate that autophagy enhances cancer cell sensitivity [18, 19]. Thus, the role and underlying mechanisms of autophagy in cancer treatment require clarification.

In our previous work, we found that MPPα-PDT activated PERK signaling in human OS (HOS) cells, although the mechanism remained unclear [19]. In the present study, the pro-survival role and underlying mechanism of PERK-eIF2α-ATF4-P21 signaling are explored, and PERK inhibitor GSK2656157 is shown to enhance the anti-tumor effects of MPPα-PDT in HOS cells via regulation of autophagy by p21. Possible factors and potential molecular mechanisms are discussed, and the results may indicate a new therapeutic strategy against OS.

## MATERIALS AND METHODS

### Cell culture

HOS cells were purchased from ATCC (USA). Dulbecco’s modified Eagle medium (Gibco, USA), supplemented with 10% fetal bovine serum (Wisent, Quebec, Canada) and 100 μg/mL pen/strep (Beyotime Biotech, Shanghai, China) was used to culture HOS cells at 37°C with 5% CO_2_.

### *In vitro* MPPα-PDT treatment

Conditions and procedures were identical to those described in our previous studies [10, 11, 19]. HOS cells were randomly grouped as follows: control (drug and LED-free); MPPα (incubation with 0.15 μM MPPα, Sigma-Aldrich); LED (drug free); and MPPα-PDT (incubation with 0.15 μM MPPα). Cells in all groups were incubated for 20 h without lighting. The cells of the MPPα-PDT and LED groups were then incubated with drug-free medium and subjected to light from LED equipment (Chongqing Jingyu Laser Technology Co., Ltd., Chongqing, China) at 630 nm wavelength and light energy density of 4.8 J/cm2 for 120 s.

### Hoechst staining

HOS cells were subjected to different treatments. Twelve hours post-treatment, they were rinsed with 1× phosphate-buffered saline (PBS, Beyotime Biotech) and fixed with 4% paraformaldehyde. Nuclei were visualized using 10 μg/mL Hoechst 33258 (Beyotime Biotech). The cells were then imaged by fluorescence microscopy (Nikon, Tokyo, Japan).

### Apoptotic assay

After HOS cells had been exposed to different treatments for 12 h, their apoptosis rates were detected by flow cytometry after Annexin V-FITC/propidium iodide (PI) (Beyotime Biotech) double staining.

### Cell cycle analysis

HOS cells were placed in six-well plates at a density of 5 × 10^6^ cells/well and subjected to different treatments for 12 h. The adherent cells and those in the basic medium and PBS were all collected, centrifuged at 1000 rpm for 5 min, and fixed overnight in PBS containing 75% ethanol at 4°C. The ethanol was removed by washing with PBS, and the intracellular RNA was digested by RNase A (200 μg/mL) for 30 min at 37°C. The cells were then stained with PI dye (50 μg/mL) for 30 min at 4°C without light before detection of the cell cycle distribution by flow cytometry.

### Fluo-4 AM staining

After undergoing different treatments for 12 h, HOS cells were incubated with Fluo-4 AM (Beyotime Biotech) at a concentration of 2 μM at 37°C for 30 min while protected from light. They were then harvested and centrifuged, and the fluorescence intensity of cytosolic-free calcium was examined by flow cytometry.

### Electron microscopy

After treatment with different interventions on 24-well culture plates, HOS cells were harvested and fixed with osmic acid and glutaraldehyde. Sections were prepared as previously reported before analysis by transmission electron microscopy (TEM).

### Immunofluorescence

Following treatment, processed HOS cells were stabilized using 4% paraformaldehyde for a duration of 20 min, made permeable with 0.1% Triton-X 100 for 5 min, and then blocked using 3% bovine serum albumin (BSA) and 0.08% glycine for a period of 50 min. Samples were incubated with polyclonal rabbit anti-phospho-PERK antibody (bs-3330R, Bioss, USA) at 1:150 overnight at 4°C and then with FITC-conjugated goat anti-rabbit secondary antibody (bs-0295G-FITC, Bioss) at 1:200 for 1 h. For sections, paraffin-embedded slides were prepared as described in section 2.14 (Histology). Sections were deparaffinized, hydrated and subjected to antigen retrieval as described previously. For immunofluorescence (IF) examination of tissues, sections were also permeabilized and blocked before incubation with anti-phospho-PERK primary antibody and goat anti-rabbit Fluor 594 secondary antibody (bs-0295G-AF594, Bioss). Then, nuclei were visualized with DAPI (Beyotime Biotech) staining, and samples were imaged by fluorescence microscopy. Fluorescence intensity was analyzed using ImageJ 1.52.

### RT-qPCR

RNA of treated HOS cells was isolated using RNAiso Plus (Cat. No. 9108, Takara, Japan), and mRNA (1 mg) was reverse transcribed using an RT-PCR assay kit (Cat. No. 6110A, RR901A, Takara). The primers used in this study were as follows:

human p21: F: 5′-GCCCGTGAGCGATGGAACTTC-3′, R: 5′-CCTGCCTCCTCCCAACTCATCC-3′; human ATF4: F: 5′-GTCTGCCCGTCCCAAACCTTAC-3′, R: 5′-TCCTGCTCCGCCCTCTTCTTC-3′; human GAPDH: F: 5′-AGGTCGGTGTGAACGGATTTG-3′, R: 5′-TGTAGACCATGTAGTTGAGGTCA-3′. GAPDH was used as a control. SYBR green/fluorescein qPCR Master Mix (Cat. No. RR820A, Takara) was used for the PCR reaction. For RT-PCR amplification, 40 cycles were carried out at 95°C for 30 s, 95°C for 5 s, and 60°C for 30 s, using a CFX96 real-time PCR system (Bio-Rad, USA). The ΔΔCt method was used to evaluate the relative expression levels of genes.

### Chromatin immunoprecipitation (ChIP)

ChIP assays were performed following the SimpleChIP^®^ protocol (Agarose Beads) (https://www.cellsignal.com/contents/resources-protocols/simplechip-sup-sup-chromatin-immunoprecipitation-protocol-(agarose-beads)/chip-agarose) by Cell Signal Technology (USA). Purified DNA was analyzed by RT-qPCR. Protein–DNA complexes were cross-linked with formaldehyde and then subjected to nuclei preparation and chromatin digestion by sonication. Anti-ATF4 antibody (11815) was used at 1:200 for ChIP with normal rabbit IgG (3900) as negative control. ChIP-grade protein G agarose beads (9007) were used to harvest DNA–protein complexes. RNase A (No 7013) and proteinase K (10012) were used to treat the precipitates. All reagents used in the ChIP assays were purchased from Cell Signaling Technology. The primer sequences used were as follows [20]: 9-1 (p21 int1): F: 5′-CCAAGAGCGCTGTCAAGAAGA-3′, R: 5′-AGGAATTCAGCTGCTGGAGG-3′. The PCR were conditions as described above.

### Short interfering RNA (siRNA) transfection

siRNAs against ATF4 and p21 and a negative control (siRNA-NC) were purchased from Hanbio Biotechnology Co., Ltd. (Shanghai, China). Lipo8000 (Beyotime Biotech) was used for siRNA transfection following the manufacturer’s instructions. Transfected HOS cells were incubated for 24 h for further examination. RT-qPCR and western blotting were used to determine relative mRNA and protein levels before and after transfection. The following siRNAs were used [21]: human p21 siRNA: 5′-GAUGGAACUUCGACUUUGU-3′; human ATF4 siRNA: 5′-GCCUAGGUCUCUUAGAUGA-3′; human p21 over-expression: LV-h-CDKN1A-E/B-F: CTAGAGGATCTATTTCCGGTGAATTCGCCACCATGTCAGAACCGGCTGGG. LV-h-CDKN1A-E/B-R CACTTAAGCTTGGTACCGAGGATCCGGGCTTCCTCTTGGAGAAGATCAG.

### Western blotting

The western blot procedure was as outlined in our previous research. In short, both HOS cells and tumors were lysed using chilled RIPA buffer (Beyotime Biotech), combined with a phosphatase and protease inhibitor cocktail (Bioss). After separation by 10% or 12% sodium dodecyl sulfate polyacrylamide gel electrophoresis, the proteins were transferred onto polyvinylidene fluoride membranes (Beyotime Biotech), which were then blocked with 5% non-fat milk for 2 h. The membranes were incubated with the following primary antibodies for a duration exceeding 12 h at a temperature of 4°C: anti-PERK (5683), anti-CHOP (2895), anti-BiP (3177), anti-ATF4 (11815), anti-LC3B (3868), anti-SQSTM1/p62 (88588), anti-Atg5 (12994) anti-cleaved caspase-3 (9664), anti-cleaved PARP (5625), anti-p21 (2947) all at 1:1000, and anti-phospho-eIF2α (3398) at 1:800, all from Cell Signaling Technology. Anti-β-actin (bs-0061R) at 1:5000, anti-phospho-PERK (bs-3330R) at 1:800, and anti-GAPDH (bs-0755R) at 1:5000 from Bioss were also used. Then, the membranes were further incubated with the corresponding HRP-labeled secondary antibodies. Detection was carried out using BiossECL Plus WB Substrate (Bioss), and band intensities were analyzed using Image Lab software.

### Xenograft model, MPPα-PDT therapy, and GSK2656157 treatment *in vivo*

The animal studies received approval from the relevant ethical board. Male nude mice, 5 weeks old, were procured from the Chongqing Medical University’s Experimental Animal Center (certificate: SCXK (Yu) 2012–0001). These animals were housed in an environment with regulated temperature and humidity, following a 12-h light/dark cycle, and were granted unrestricted access to food and water. Xenograft model generation and MPPα-PDT treatment were done as described previously [9]. Briefly, nude mice were anesthetized and injected subcutaneously in the back with 100 μL PBS containing 1 × 10^6^ HOS cells. When tumors reached 8 ± 1 mm in diameter, animals were split into six groups (three mice in each group): the control, MPPα, LED, GSK2656157 (S7033, Selleck, USA), MPPα-PDT, and MPPα-PDT+GSK2656157 groups. MPPα was injected into the tail vein at 15 mg/kg, and the animals were held in a dark room for 18 h to allow biodistribution of MPPα into the tumor. They were then exposed to 630 nm light at 120 J/cm2, for 20 min every other day, for 10 days. GSK2656157 was prepared in a solution composed of 0.5% hydroxypropyl methyl cellulose and 0.1% Tween-80 in water with a pH of 6.75 and orally administered at 50 mg/kg twice daily [22]. Tumor volume was monitored every 4 days for 30 days after treatment and calculated as follows: tumor volume = (length × width2)/2.

### Histology

Fresh tissue was fixed with 4% formaldehyde for more than 24 h, dehydrated with an increasing alcohol concentration gradient, and embedded in paraffin. The wax block was sliced to a thickness of 1 mm. Slides were then incubated in hematoxylin and eosin.

### Immunohistochemistry (IHC)

Slices were dried in an oven at 60°C and dewaxed by soaking in different concentrations of xylene and ethanol. The sections were placed in an antigen repair solution prepared in advance. Then, 3% H_2_O_2_ was added to remove endogenous peroxidase, and 1% BSA was added for blocking. Primary antibody was added to the tissue, with incubation overnight at 4°C, followed by further incubation with secondary antibody at 37°C. Hematoxylin was used to stain the nuclei. Finally, the sections were observed and recorded under a microscope at a 200X magnification. IHC quantification was done using IHC Profiler according to the following levels: 4, high positive; 3, positive; 2, low positive; 1, negative.

### TUNEL assay

Slices were soaked in xylene and different concentrations of ethanol (twice with 100% for 3 min, then with 85% for 3 min and 75% for 3 min) before treatment with proteinase K (0.5%) for 25 min at 37°C and Triton-X-100 for 20 min at room temperature. A TUNEL assay kit (Cat. No. 11684817910, Roche Diagnostics, USA) was used according to the manufacturer’s instructions. Briefly, the TUNEL reaction mixture (TdT + dUTP mixed at a 1:9 ratio) was prepared according to the instructions, added to sections, and allowed to react for 2 h at 37°C. Nuclei were stained with DAPI at room temperature for 10 min. Apoptotic cells were examined under a fluorescence microscope.

### GFP-LC3 transfection

HOS cells, at a quantity of 1 × 10^5^ per well, were placed in a confocal dish. Following a 24-h culture period post-treatment, adenovirus GFP-LC3 (Hanbio, Shanghai, China) was introduced, and the cells were left for another 24 h. Visual data were acquired using laser confocal microscopy (TCS-SP5, Leica, Germany). The count of green fluorescent specks was considered to indicate the extent of autophagy.

### Mitochondrial membrane potential assay

A JC-1 staining kit (Beyotime Biotech, China) was used to evaluate the mitochondrial membrane potential (ΔΨm) of treated HOS cells. Post-treatment, a staining buffer was introduced and left for 20 min at a temperature of 37°C. The staining of HOS cells was observed and assessed through fluorescence microscopy and flow cytometry.

### Data analysis and statistics

Data are presented as the mean ± standard deviation. Data measurements were analyzed using Student’s *t*-test or analysis of variance in GraphPad Prism 7. Every experiment was conducted a minimum of three times. A value of *p* < 0.05 was deemed to indicate statistical significance.

### Data availability

The datasets used or analyzed during the current study are available from the corresponding author on reasonable request.

## RESULTS

### MPPα-PDT induces apoptosis and cycle arrest

**Figure 1:**
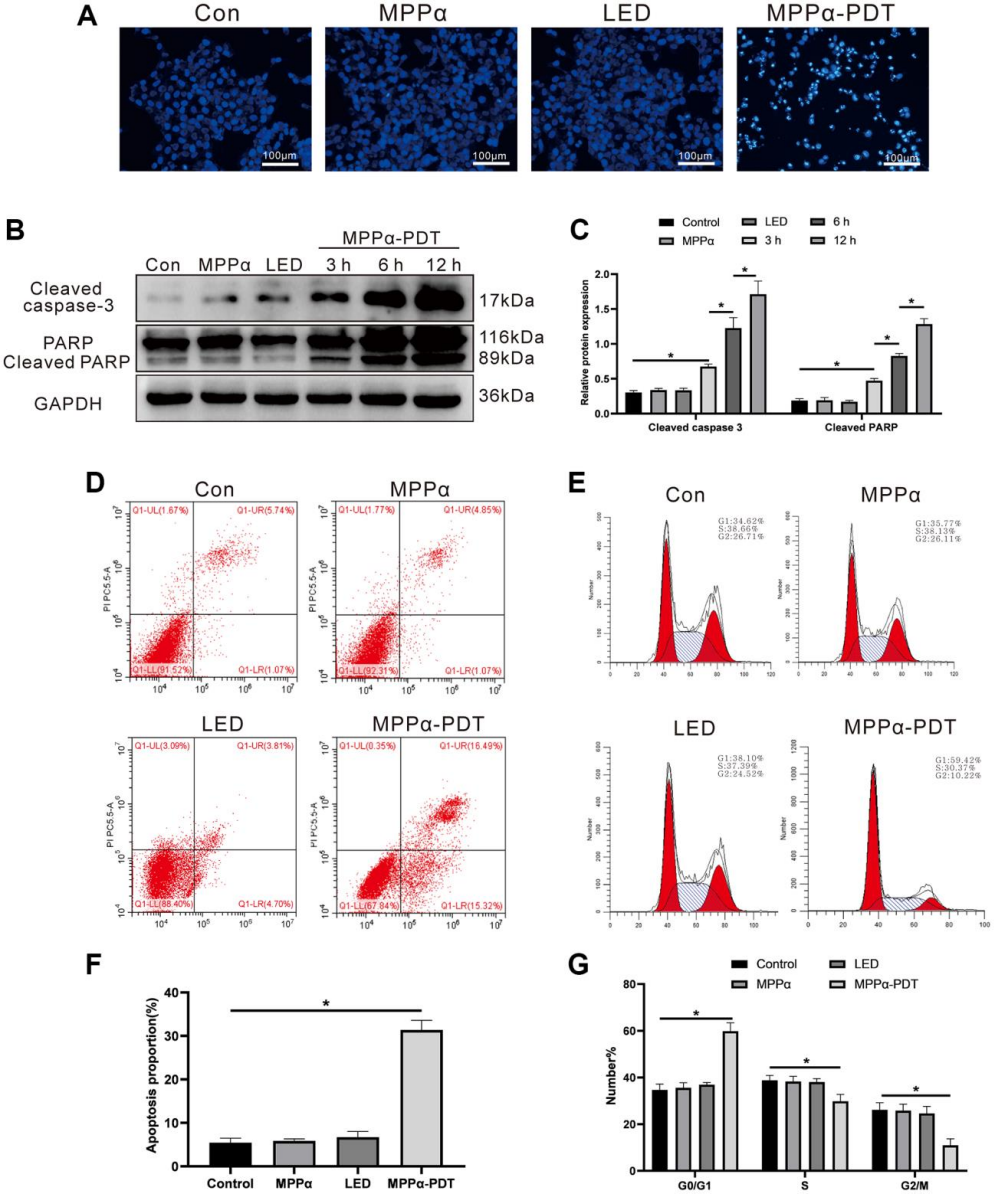
**MPPα-PDT induces HOS cells cycle arrest and apoptosis.** (**A**) HOS cells nuclei were examined for apoptotic morphological changes under a fluorescence microscope (magnification: ×200) after MPPα-PDT treatment for 12 h. (**B**, **C**) Cells were harvested after MPPα-PDT treatment for 3, 6, and 12 h and western blot used to evaluate cleaved caspase-3 and cleaved PARP levels. (**D**–**F**) Apoptosis was determined by flow cytometry. (**E**–**G**) Cell cycle distribution was analyzed using flow cytometry. ^*^P < 0.05 vs. control group. All data represent the mean ± SD of 3 independent experiments.

To explore the anti-tumor mechanism of MPPα-PDT, we examined a series of apoptotic indicators. HOECHST staining revealed apoptotic morphological changes, including nuclear fragmentation and chromatin condensation, following 12 h MPPα-PDT treatment (Figure 1A). No marked changes were observed in the control, MPPα and LED groups. Concurrently, we investigated apoptosis-related proteins, including cleaved caspase-3 and cleaved PARP, using western blot analysis. Levels of cleaved caspase-3 and cleaved PARP both increased in response to MPPα-PDT treatment at 3, 6, and 12 h (*P* ≤ 0.01, Figure 1B, 1C). Further, we used flow cytometry to evaluate the influence of MPPα-PDT on the cell cycle and apoptosis (*P* ≤ 0.01, Figure 1D–1F). Following MPPα-PDT treatment, a significant increase was observed in the proportion of HOS cells arrested in the G0/G1 phase, accompanied by notable reductions in the percentages of cells residing in the S and G2/M phases (*P* ≤ 0.05, Figure 1E–1G). There were no discernible variations in cell cycle distribution in the remaining three groups (*P* ≥ 0.05, Figure 1E–1G). The G0/G1 phase denotes the initial stage of cellular division wherein cells undergo growth and prepare for entry into the S phase of DNA replication. By impeding progression through the cell cycle during this specific phase, a multitude of effects can be elicited, including inhibition of cellular proliferation, heightened treatment sensitivity, induction of apoptosis, suppression of DNA replication, and reduction in relapse. These findings suggest that MPPα-PDT can trigger G0/G1 cell cycle arrest and apoptosis in HOS cells. No noticeable differences in the distribution of cell cycle phases were found among the other three groups (*P* ≥ 0.05, Figure 1E–1G). This evidence points towards an ability of MPPα-PDT ability to induce G0/G1 cycle arrest and apoptosis in HOS [7, 8, 11].

### MPPα-PDT triggers ER stress and PERK-eIF2α-ATF4 signaling pathway activation

**Figure 2:**
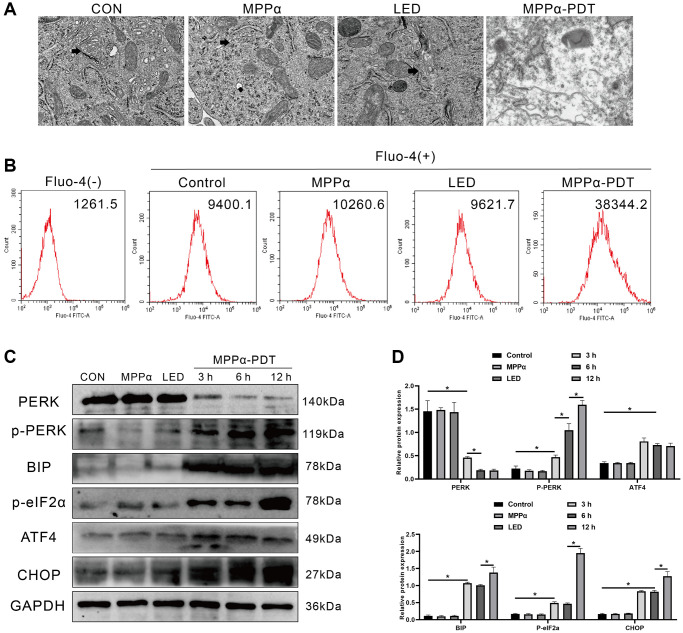
**ER stress and PERK-eIF2α-ATF4 signaling pathway activation are induced by MPPα-PDT in HOS cells.** (**A**) ER morphology was observed by TEM (magnification, ×20000). (**B**) Intracellular Ca^2+^ concentration was quantified by flow cytometry using Fluo-4-AM probe. (**C**, **D**) MPPα-PDT treated cells were harvested after 3, 6, and 12 h and PERK, p-PERK, BIP, p-eIF2α, ATF4, and CHOP, levels determined by western blot. Data are shown as mean ± SD of 3 independent experiments. ^*^P < 0.05 vs. control group.

PDT can produce large amounts of ROS, which may disrupt ER REDOX regulation and homeostasis, leading to ERS and an increase in misfolded/unfolded proteins. Therefore, we preliminarily investigated whether ERS is involved in the potential mechanism of MPP-α-PDT anti-tumor by transmission electron microscopy (TEM). As we observed, the ER of HOS cells showed significant morphological damage after MPPα-PDT treatment, indicating the occurrence of ERS (Figure 2A). The ER is a dynamic organelle involved in various cellular functions, including the regulation of lipid metabolism, calcium storage, and protein balance. During stress, calcium ions are released from the ER into the cytoplasm, initiating a cascade of cell signaling pathways and physiological responses. Therefore, it is crucial to detect calcium ion leakage during ER stress to gain insights into the signal transduction network and physiological response within the cell. As shown in Figure 2B, relative to the other three groups, intracellular Ca^2+^ concentration ((Ca^2+^)i) was much higher in the MPPα-PDT treatment group. Western blotting was used to determine whether PERK-eIF2α-ATF4 signaling was activated by MPPα-PDT. The data revealed a significant increase in PERK-eIF2α-ATF4 signaling-related proteins, including p-PERK, BIP, p-eIF2α, ATF4, and CHOP, following MPPα-PDT treatment (*P* ≤ 0.05, Figure 2C, 2D). These findings indicate that MPPα-PDT induces characteristic manifestations of ER stress such as disrupted ER morphology, elevated intracellular calcium concentration ((Ca^2+^)i), and activated PERK-eIF2α-ATF4 signaling.

### Autophagy can be triggered by MPPα-PDT

**Figure 3:**
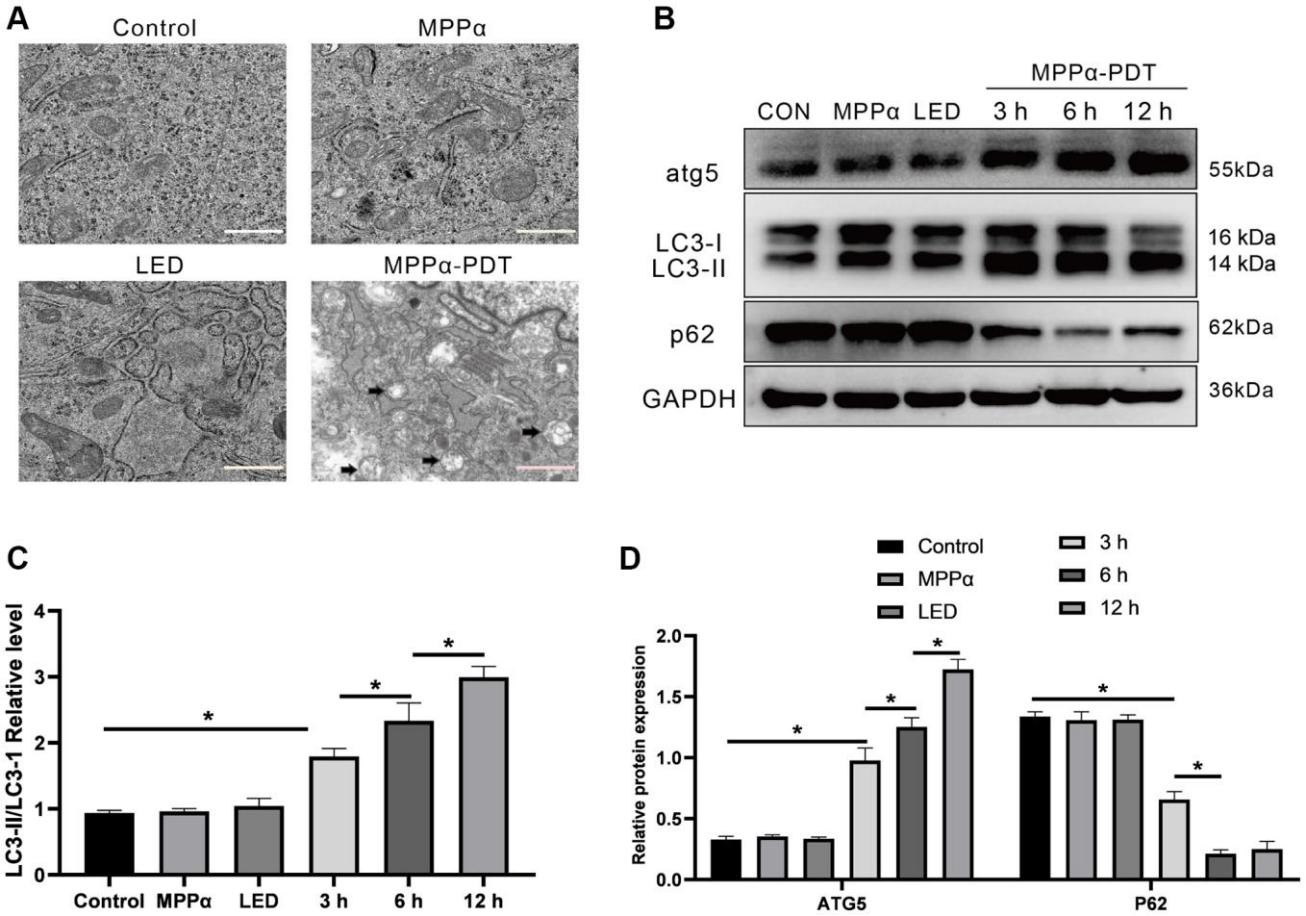
**Autophagy can be triggered by MPPα-PDT.** (**A**) Transmission electron microscopy (magnification, ×20000) was used to observe autophagosomes upon autophagy induction by MPPα-PDT after 12 h. (**B**–**D**) Cells were harvested 3, 6, and 12 h after MPPα-PDT treatment and protein ATG5, LC3-II/LC3-I, and p62 were determined by western blot. Data are shown as mean ± SD of 3 independent experiments. ^*^P < 0.05 vs. control group.

Autophagy is a highly conserved decomposition process regulated by genes. Generally, it is a regulatory mechanism by which cells resist adverse stress or external damage. Thus, we tested whether autophagy was involved in the mechanism of MPPα-PDT. First, abundant autophagosomes in the MPPα-PDT group were observed through TEM analysis (black arrows, Figure 3A). Moreover, western blot analysis revealed elevated LC3-II/LC3-I ratios and ATG5 levels, as well as significantly reduced p62 levels, in HOS cells after MPPα-PDT treatment (*P* ≤ 0.05, Figure 3B–3D). Taken together, these results indicate that MPPα-PDT can induce autophagy.

### Blocking PERK can inhibit autophagy triggered by MPPα-PDT and augment the anti-tumor efficacy of MPPα-PDT in HOS cells

**Figure 4:**
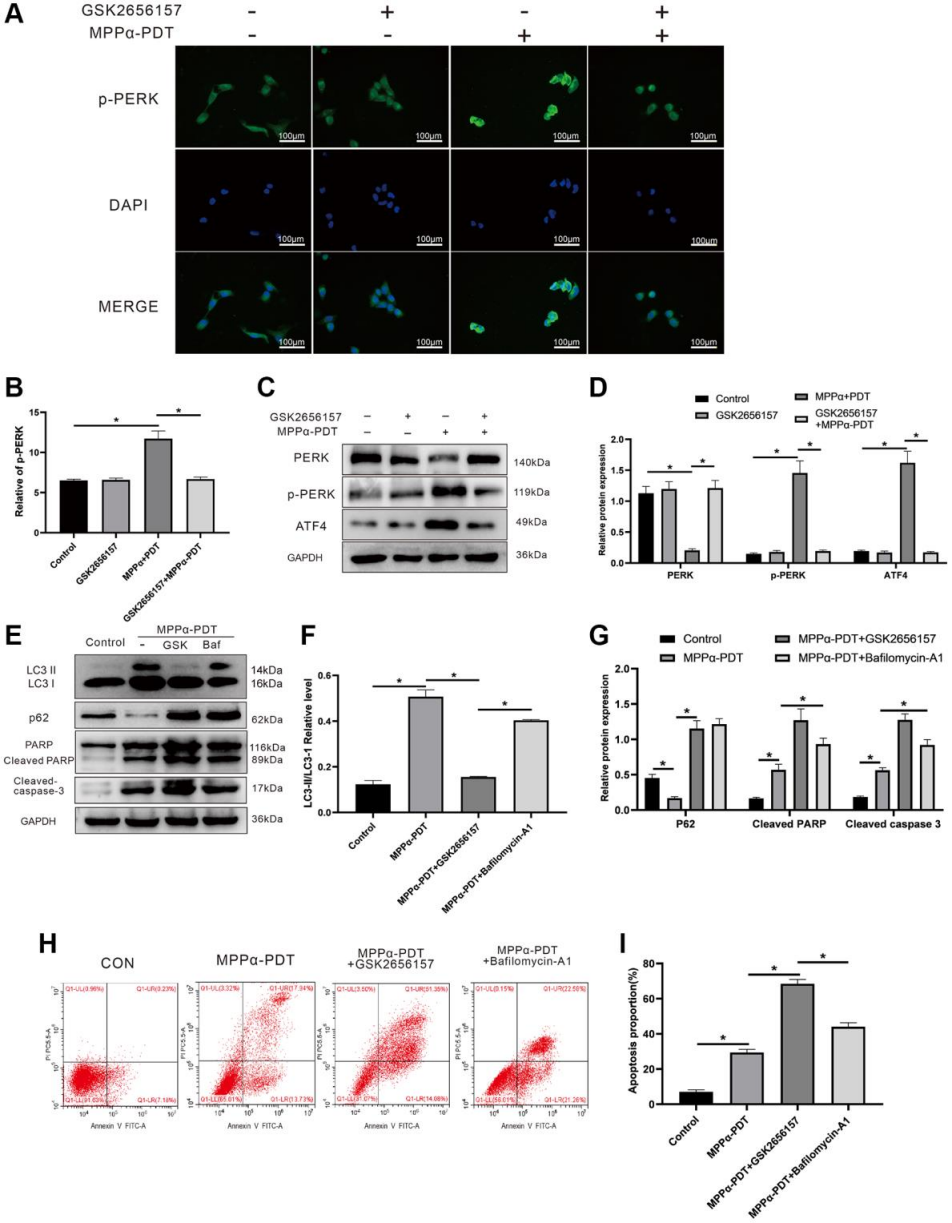
**Inhibiting PERK may suppress autophagy induced by MPPα-PDT, and enhance the anti-tumor ability of MPPα-PDT in HOS cells.** HOS cells in MPPα-PDT+GSK2656157 group were pretreated with 5 mM GSK2656157 for 1 h before MPPα-PDT treatment. HOS cells in MPPα-PDT + Bafilomycin A1 group were pretreated with 100 nM bafilomycin A1 for 2 h before MPPα-PDT treatment. (**A**, **B**) Immunofluorescence analysis of p-PERK levels (magnification: ×400). (**C**–**G**) After indicated treatments, cells were harvested and PERK, p-PERK, ATF4, LC3-II/LC3-I, p62, cleaved caspase-3, and cleaved PARP levels determined by western blot. (**H**, **I**) Apoptotic rate was examined by flow cytometry. Data are shown as mean ± SD of 3 independent experiments. ^*^P < 0.05.

Having demonstrated that ER stress and autophagy could be induced by MPPα-PDT, we further explored this relationship. To achieve complete inhibition of the PERK pathway, we pretreated HOS cells with PERK inhibitor GSK2656157 (5 mM; Selleck Chemicals, Cat. No. S7033) for 2 h. Increased p-PERK fluorescence intensity was observed by IF after MPPα-PDT treatment (*P* ≤ 0.05 Figure 4A, 4B). Western blot analysis showed that MPPα-PDT increased p-PERK and ATF4 levels and reduced PERK levels (*P* ≤ 0.01, Figure 4C, 4D), demonstrating that the activation of PERK induced by MPPα-PDT could be reversed by GSK2656157. Moreover, the anti-tumor effect of MPPα-PDT was markedly strengthened after GSK2656157 treatment, as reflected by an increase in levels of apoptotic proteins. Changes in the expression of LC3-II/I and p62 indicated successful autophagy blocking after bafilomycin A1 pretreatment, with similar inhibitory effects observed for GSK2656157 (*P* < 0.05, Figure 4E–4G). These findings were supported by the flow cytometry results (*P* ≤ 0.05 Figure 4H, 4I). In summary, our results indicate that PERK-eIF2α-ATF4 signaling blockade may suppress protective autophagy and other pro-survival mechanisms.

### Targeting ATF4 increases the sensitivity of PDT by inhibiting autophagy

**Figure 5:**
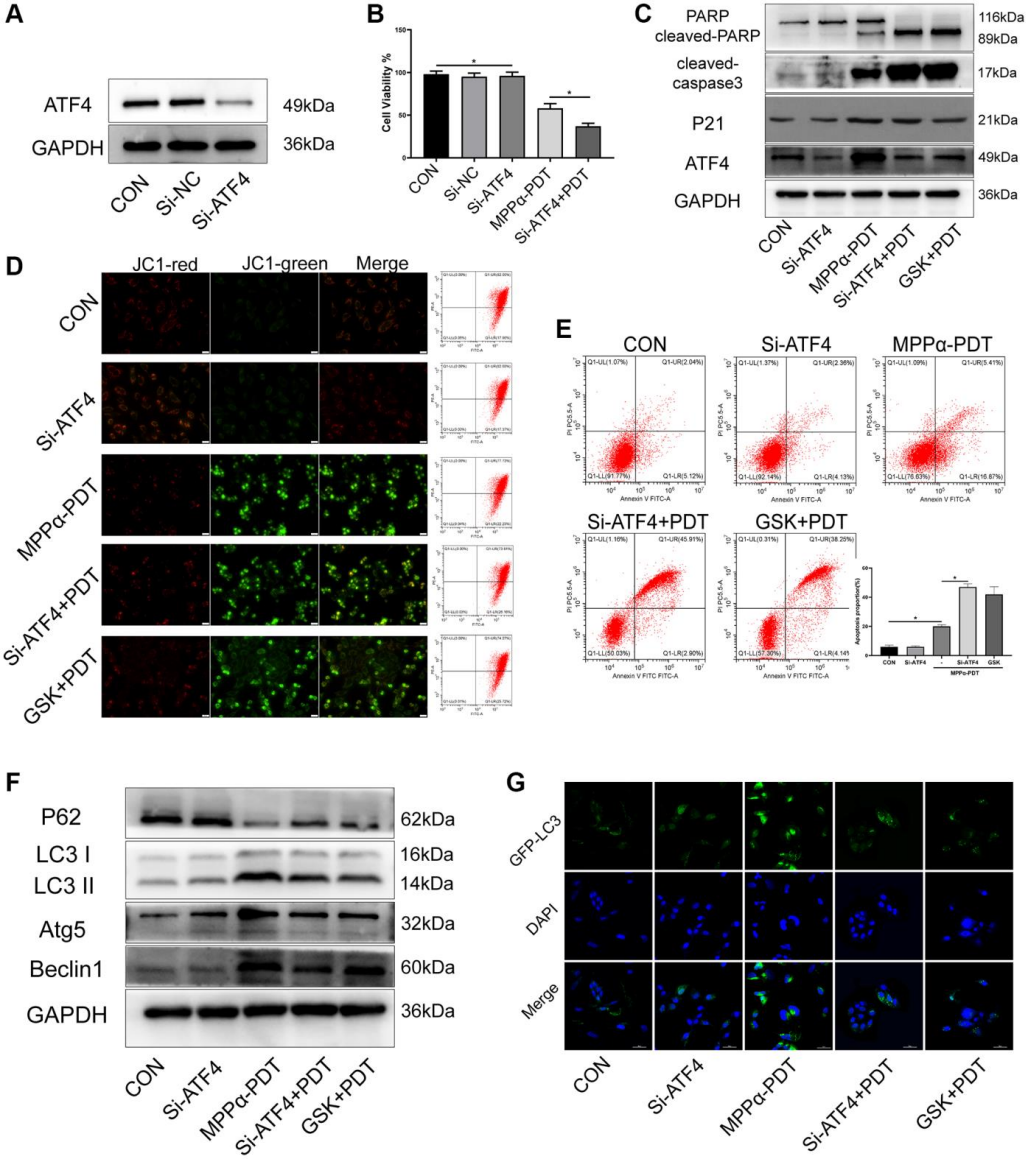
**Targeting ATF4 increases the sensitivity of PDT by inhibiting autophagy.** HOS cells in MPPα-PDT+GSK2656157 group were pretreated with 5 mM GSK2656157 for 1 h before MPPα-PDT treatment. HOS cells were transfected with siRNAs and then treated with MPPα-PDT. (**A**) Protein ATF4 or mRNA ATF4 was detected by western blot and represents the transfection efficiency. (**B**) Cell viabilities were detected by CCK-8 after treated with SiRNA-ATF4 (Si-ATF4), SiRNA-negative control (Si-NC), MPPα-PDT and group MPPα-PDT combined with Si-ATF4. (**C**) Following indicated treatments, cells were harvested and ATF4, p21, PARP and cleaved caspase-3 levels determined by western blot. (**D**, **E**) Apoptotic rate or JC-1 stain was examined by flow cytometry or fluorescence microscope (×200). (**F**) Following indicated treatments, cells were harvested and LC3, P62, Atg5 and Beclin1 levels were determined by western blot. (**G**) Adenovirus-GFP-LC3 was transfected into the HOS cells after treated, the LC3 fluorescent particles were observed by laser confocal microscope (×400). ^*^P < 0.05 vs. control group, data are shown as mean ± SD of 3 independent experiments.

ATF4, a downstream effector of the PERK pathway, mediates the adaption of cells to harmful conditions such as ER stress, and modulates expression of various pro-survival factors including p21. We used western blotting to determine whether induction of p21 expression by MPPα-PDT requires ATF4. Anti-ATF4 siRNA significantly suppressed ATF4 expression and cell viability (*P* ≤ 0.01, Figure 5A, 5B, Supplementary Figure 1). ATF4 and p21 levels were upregulated by MPPα-PDT, whereas ATF4 depletion suppressed p21 induction by MPPα-PDT. PERK signaling blockade by GSK2656157 pretreatment suppressed ATF4 and p21 induction by MPPα-PDT. Apoptosis proteins PARP and cleaved-caspase 3 were activated by GSK2656157 or siRNA-ATF4 under MPPα-PDT treatment (*P* ≤ 0.05, Figure 5C, Supplementary Figure 2). Levels of apoptosis were detected by flow cytometry and JC-1 staining (Figure 5D, 5E). Autophagy was found to be activated by MPPα-PDT but inhibited by GSK2656157 or siRNA-ATF4, as indicated by the levels of autophagy proteins including LC3, P62, and Beclin 1, and of GFP-LC3 fluorescent particles (Figure 5F, 5G, Supplementary Figure 3). Thus, as the key effector of PERK pathway, ATF4 is a potential target that could be used to enhance the efficacy of MPPα-PDT.

### The pro-survival effect of p21 is achieved by regulation of autophagy after MPPα-PDT

**Figure 6:**
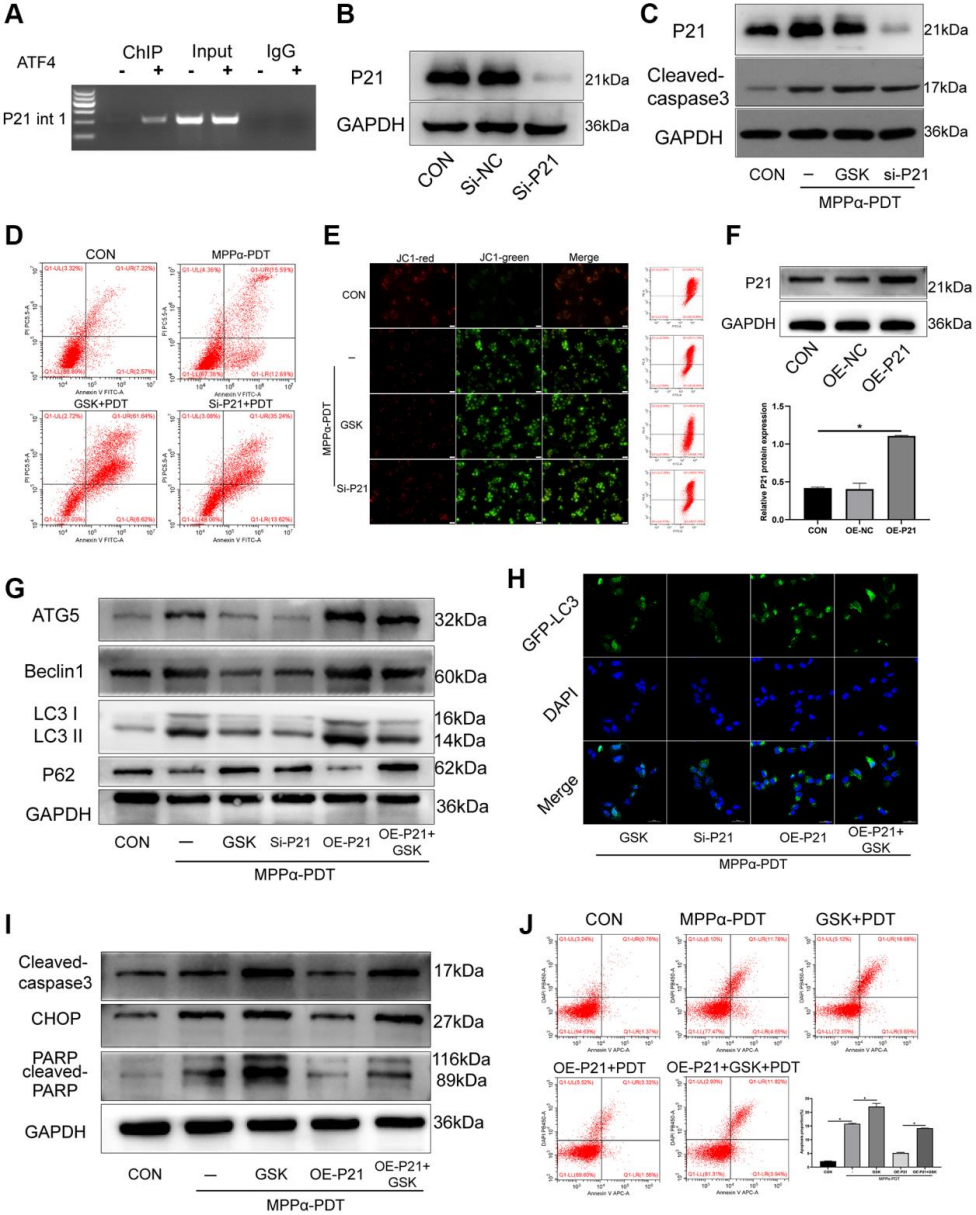
**The pro-survival effect of P21 is achieved by regulating autophagy after MPPα-PDT.** (**A**) ChIP analysis was used to detect ATF4 binding to the first intron of p21. HOS cells were transfected with siRNAs and then treated with MPPα-PDT. (**B**) Protein P21 were detected by western blot to represent the transfection efficiency. (**C**) Following indicated treatments, cells were harvested and P21 and cleaved caspase-3 levels were determined by western blot. (**D**, **E**) Apoptotic rate or JC-1 stain were examined by flow cytometry or fluorescence microscope (×200). (**F**) Protein P21 was detected by western blot after transfected by lentivirus-overexpress P21. (**G**) Following indicated treatments, cells were harvested and LC3, P62, Atg5 and Beclin1 levels were determined by western blot. (**H**) Adenovirus-GFP-LC3 was transfected into the HOS cells after treated, the LC3 fluorescent particles were observed by laser confocal microscope (×400). (**I**) Protein CHOP, cleaved-caspase3 were detected by western blot. (**J**) Apoptotic rate was examined by flow cytometry. ^*^P < 0.05 vs. control group, data are shown as mean ± SD of 3 independent experiments.

Cyclin-dependent kinase inhibitor (CDKI/P21) is the target gene encoded by ATF4, and promotes cell survival under ER stress. Past studies have shown that ATF4 binds in the first intron of p21 at +710 to +724 (GCGCTGAGGTCAGCG) to induce p21 expression [20]. Here, ChIP analysis showed that ATF4 bound to the first intron of p21 after MPPα-PDT treatment (Figure 6A). Next, we sought to elucidate the role of p21 in PERK signaling activation. To this end, we used western blotting to determine the influence of p21 on the survival of HOS cells. Compared with the MPPα-PDT group, cells in the p21-silencing group had increased cleaved caspase-3 levels. Similarly, PERK inhibition with GSK2656157 pretreatment reduced p21 levels but upregulated cleaved capase-3, relative to levels in the MPPα-PDT group (*P* ≤ 0.05, Figure 6B, 6C, Supplementary Figures 4 and 5). Apoptosis analysis by flow cytometry revealed that p21 depletion enhanced the cytotoxicity of MPPα-PDT (*P* ≤ 0.05, Figure 6D, 6E). These data indicate that induction of p21 expression by ATF4 may promote survival of HOS cells. PERK-eIF2α-ATF4 signaling inhibition enhanced MPPα-PDT cytotoxicity by reducing p21 expression. Next, p21 was overexpressed using a lentivirus vector, and the transfection efficiency was detected by western blotting (*P* ≤ 0.05, Figure 6F). Autophagy was found to be activated by p21 overexpression after treatment with MPPα-PDT (*P* ≤ 0.05, Figure 6G, 6H, Supplementary Figure 6). Moreover, apoptosis proteins including PARP, CHOP, and cleaved-caspase 3 were inhibited by p21 overexpression after MPPα-PDT treatment (*P* ≤ 0.05, Figure 6I, 6J, Supplementary Figure 7). Briefly, these results indicate that the pro-survival effect of p21 is achieved by regulation of autophagy after MPPα-PDT treatment.

### GSK2656157 enhances anti-tumor activity of MPPα-PDT by inhibiting PERK pathway *in vivo*

**Figure 7:**
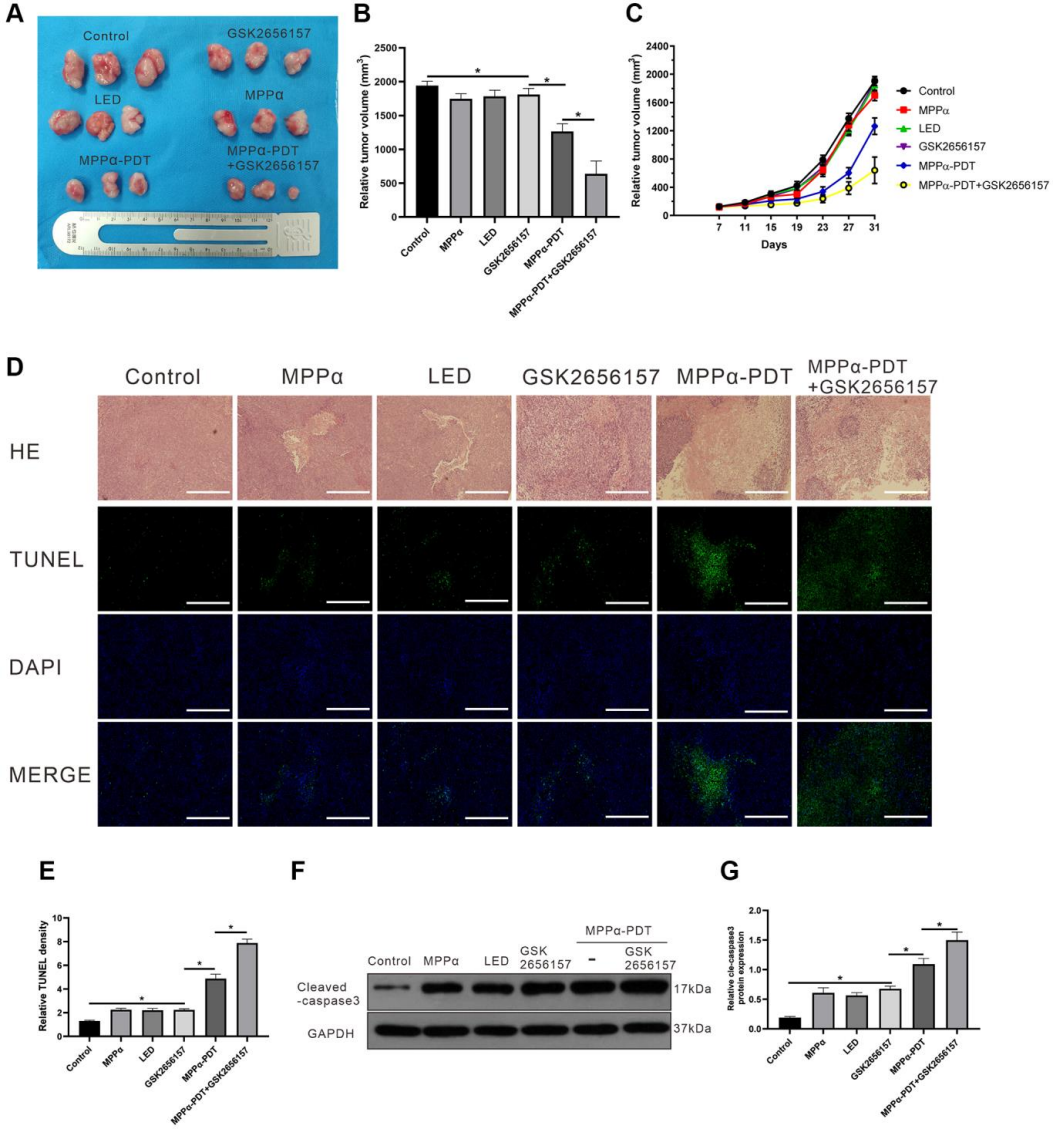
**GSK2656157 enhances the antitumor activity of MPPα-PDT by inhibiting PERK pathway *in vivo*.** HOS tumor-bearing mice were treated with MPPα (15 mg/kg), LED (120 J/mm2), GSK2656157 (30 mg/kg) and MPPα-PDT (LED following MPPα, as described in Materials and Methods). (**A**, **B**) After 31 days, tumors were collected. Tumor volume was determined as described in methods. (**C**) Tumor volume change curves. (**D**, **E**) After treatments, tumor necrosis and apoptosis were analyzed by H&E and TUNEL analysis, respectively (magnification, ×100). (**F**, **G**) After treatments, tumors apoptosis was assessed by cleaved caspase-3 western blot analysis. Data are shown as mean ± SD of 3 independent experiments. ^*^P < 0.05.

Next, we examined whether GSK26556157 enhanced MPPα-PDT efficacy against HOS *in vivo* by evaluating tumorigenicity in a xenograft mouse tumor model following various treatments. Tumor volume was monitored every 4 days, and tumor size growth curves were drawn (*P* ≤ 0.05, Figure 7A–7C). Treatment with MPPα-PDT decreased the proliferation of tumors compared with the control group, and MPPα-PDT in combination with GSK2656157 enhanced anti-tumor effects relative to MPPα-PDT alone. Hematoxylin and eosin staining analysis of tumor tissues revealed large necrosis areas following MPPα-PDT treatment, which were enhanced by combination treatment with GSK2656157 (*P* ≤ 0.05; Figure 7D). TUNEL staining revealed elevated apoptosis in the MPPα-PDT and MPPα-PDT+GSK2656157 treatment groups, with a greater increase in the latter (*P* ≤ 0.05, Figure 7D, 7E). To establish whether GSK2656157 treatment enhances the cytotoxicity of MPPα-PDT, we used western blotting to assess cleaved caspase-3 expression. The results showed that cleaved caspase-3 expression levels were elevated by MPPα-PDT and were higher still in the MPPα-PDT+GSK2656157 group (*P* ≤ 0.05, Figure 7F, 7G).

### p-PERK *in vivo* levels following treatments

**Figure 8:**
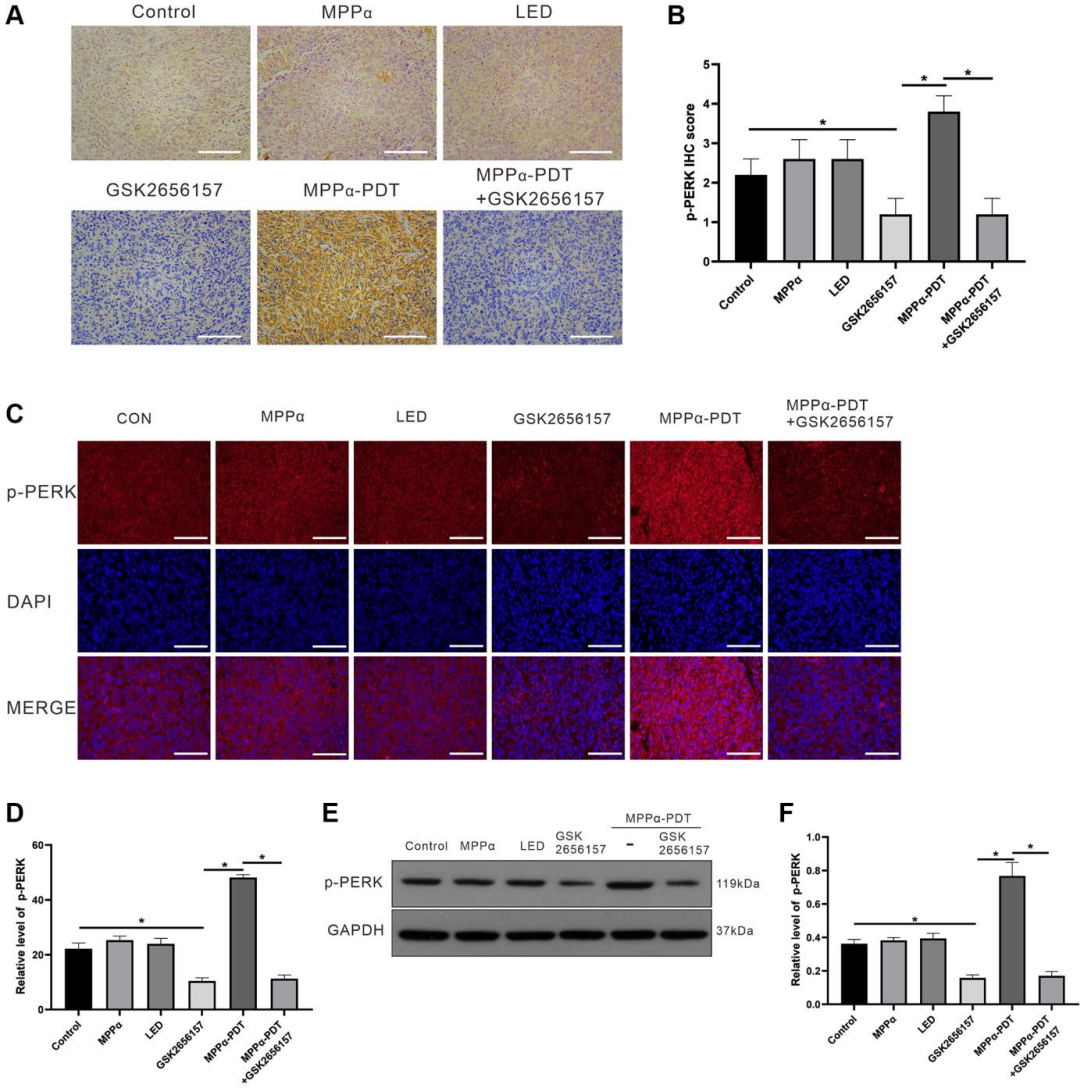
**p-PERK *in vivo* levels upon indicated treatments.** (**A**, **B**) IHC analysis of p-PERK levels in tumor sections (magnification, ×200). (**C**, **D**). Fluorescence analysis of p-PERK levels in tumor sections (magnification, ×400). (**E**, **F**) Following indicated treatments, tumor tissues were analyzed by western blot for p-PERK levels. Data are shown as mean ± SD of 3 independent experiments. ^a^*P* < 0.01 vs. control group; ^b^*P* < 0.01 vs. MPPα-PDT group.

p-PERK is both a sign of PERK signaling activation and a target of GSK2656157 inhibition. IHC analysis revealed lower positive p-PERK levels in the control, MPPα, and LED groups compared with those in the MPPα-PDT group. On the contrary, p-PERK levels were markedly reduced by GSK2656157 alone and by MPPα-PDT+GSK2656157 (*P* ≤ 0.01, Figure 8A, 8B). Analysis of p-PERK levels by IF gave similar results to those obtained with IHC. MPPα-PDT significantly elevated p-PERK expression, whereas GSK2656157 reduced it alone or in combination with MPPα-PDT (*P* ≤ 0.01, Figure 8C, 8D). Western blotting results showed that relative to controls, p-PERK levels were elevated by MPPα-PDT but suppressed by GSK2656157 alone or in combination with MPPα-PDT (*P* ≤ 0.01, Figure 8E, 8F). These results indicate that MPPα-PDT has anti-tumor effects *in vivo*, which are enhanced by PERK inhibition with GSK2656157.

## DISCUSSION

Among primary bone cancers, osteosarcoma is the most common, with a bimodal age distribution of onset, with the first peak occurring in patients aged 10–14 years and the second peak occurring in patients over 65 years, with about 25% of cases occurring in adults aged 20–59 years [23]. However, the underlying tumor biology of osteosarcoma in children and adults is significantly heterogeneous, including: chromosome instability [24], abnormal mitotic signaling pathways and cell cycle checkpoints [25], telomere dysregulation [26], and miRNA expression [27] et al. Compared to pediatric osteosarcomas, adult osteosarcomas are more likely to be secondary osteosarcomas associated with prior radiation therapy or, in some cases, Paget’s disease (a disorder of bone metabolism characterized by overactivity of bone cells and the formation of bone matrix by osteoblasts in a faster and disordered manner, producing their characteristic “Mosaic” pattern [23]). Radiation exposure is another cause of secondary osteosarcoma in adults, typically occurring many years after the exposure. Osteosarcoma is also the most common bone tumor caused by radiation, accounting for 50–60% of cases [28]. Secondary osteosarcoma is associated with increased morbidity and mortality compared to primary bone tumors. Therefore, secondary osteosarcoma may result in poor overall prognosis estimates for adult osteosarcoma. The killing mechanism of PDT involves the activation of photosensitizers to generate photochemical reactions via external light of a specific wavelength. This causes production of abundant cytotoxic ROS, resulting in oxidative damage to tumor cells [29]. MPPα, a chemical substance extracted from chlorophyll with low toxic side-effects, is a second-generation photosensitizer. MPPα-PDT has been proven to have high-efficiency killing effects in various tumors [30, 31]. Similar to previous studies [6–8, 10], we observed apoptosis in HOS cells after MPPα-PDT, as reflected by fragmented nuclei or increased levels of apoptotic proteins.

Owing to early lung metastasis and chemotherapy resistance, OS has a poor prognosis. To escape death, tumor cells deploy various signaling pathways to relieve cellular stress [32, 33]. Recent studies indicate that ER stress in response to anti-tumor therapy may disrupt ER function, thereby affecting protein synthesis, disturbing intracellular Ca^2+^ homeostasis, and contributing to therapy resistance [34]. This may rely on the regulation of UPR signaling pathways (PERK, IRE1α, and ATF6). During ER stress, PERK can be released from GRP78, resulting in PERK kinase dimerization and autophosphorylation. In turn, this may lead to eIF2α phosphorylation, which may inhibit protein synthesis and CHOP/caspase-12 activation [19]. PERK signaling may be activated by various anti-tumor strategies to enhance survival of tumor cells. Ang Zhao et al. [35] showed that inhibition of OS cell proliferation by β-elemonic acid was accompanied by significant PERK signaling activation. Similarly, we found that PERK signaling could be also activated by MPPα-PDT, as reflected by an increase in expression of PERK-related proteins and concomitant ER stress events. Here, we sought to establish whether MPPα-PDT-induced PERK signaling is pro-death or pro-survival in HOS cells. We found that the PERK pathway could protect HOS cells from the cytotoxic effects of MPPα-PDT and that its inhibition could overcome the survival-promoting effects, enhancing the sensitivity of HOS cells to MPPα-PDT. Consistent with previous research, their findings demonstrate that disabling PERK signaling hinders the clonogenic and growth abilities of CML cells and amplifies apoptosis triggered by imatinib [34].

Autophagy is a major regulator of cellular metabolism, growth, and nutrient recycling and is required for tumor survival. Autophagy flux correlates with metabolic or therapeutic challenges. Previously, we showed that MPPα-PDT induced autophagy in MG63 cells, a HOS cell line [7]. Similarly, autophagic vacuoles were observed in HOS cells after MPPα-PDT treatment, and autophagic flux analysis revealed elevated expression of autophagy-related proteins after MPPα-PDT. Taken together, these data suggest that MPPα-PDT enhances autophagosome formation and autophagy in HOS cells. Autophagy is mainly involved in cellular survival under stress conditions. However, some studies have linked it to cell death paradoxically. To determine the role of autophagy during the MPPα-PDT treatment, we used an inhibitor, bafilomycin A1, to suppress cellular autophagy. Apoptosis was enhanced markedly by bafilomycin A1 pretreatment combined with MPPα-PDT, as reflected by the results of our analysis of proapoptotic factors and flow cytometry. PERK-eIF2α-ATF4 signaling shares some underlying molecular mechanisms on inducing autophagy. PERK-eIF2α-ATF4 signaling also comprises a unique molecular network in autophagy regulation upstream of mTORC1 [36, 37]. To determine whether autophagy could be mediated by PERK-eIF2α-ATF4 signaling, we inhibited PERK autophosphorylation using inhibitor GSK2656157. Reduced expression of PERK downstream factors (p-PERK and ATF4) was observed, resulting in autophagic flux attenuation in HOS cells. Given the protective role of autophagy in HOS cells after MPPα-PDT, we hypothesized that pro-survival autophagy might be mediated by PERK-eIF2α-ATF4 signaling, which would be suppressed by PERK inhibitor GSK2656157. Our data show that apoptosis levels were much higher when MPPα-PDT was combined with GSK2656157 relative to MPPα-PDT alone or MPPα-PDT + bafilomycin A1, there were also higher expression levels of cleaved caspase-3 and cleaved PARP. Ji et al. [38] found that PERK depletion could suppress protective autophagy while enhancing stress sensitivity, potentially leading to apoptosis. These results indicate that PERK inhibition may suppress the protective effects of other mechanisms, as well as autophagy mediated by PERK signaling.

Another important theme that emerged from the data was that other protective mechanisms induced by PERK signaling may contribute to survival of HOS cells. ATF4 has a dual function in life–death decisions during treatment. Long-term, intensive stress may turn ATF4 into a pro-apoptotic factor that activates caspase-12-dependent, ER-stress-mediated apoptosis and elevated CHOP levels. On the contrary, ATF4 contributes to stress relief and survival through coping cytoprotective genes [39]. In the present work, we found that apoptosis proteins were activated by GSK2656157 or siRNA-ATF4 under treatment with MPPα-PDT. Apoptosis levels were detected by flow cytometry or JC-1 staining. As a key molecule in the PERK pathway that mediates autophagy, ATF4 can induce the formation of an ATG12-ATG5-ATG16 complex, which is associated with ATG8 and is involved in the formation of autophagosomes. Simultaneously, ATF4 activation induces increased downstream CHOP expression and inhibits the expression of the mTORC1 pathway, thereby enhancing autophagy activity [40]. We also found that autophagy was activated by MPPα-PDT but inhibited by GSK2656157 or siRNA-ATF4. Thus, as the key effector of the PERK pathway, ATF4 is a potential target that could be used to enhance the efficacy of MPPα-PDT.

Cyclin-dependent kinase inhibitor (p21; CDKI) is a target gene encoded by ATF4 and plays a role in promoting cell survival under ER stress. Here, the ChIP assay revealed that p21 intron 1 bears ATF4-responsive elements that bind to ATF4. ATF4 silencing suppressed p21 expression levels after MPPα-PDT treatment, consistent with past findings [20, 41]. Our findings confirm the pro-survival role of p21, which could be suppressed by PERK signaling inhibition. P21 expression was increased by elevated ATF4 under ER stress, whereas p21 silencing enhanced apoptosis after MPPα-PDT treatment in HOS cells. These results indicate a pro-survival role of p21. Furthermore, it has been established that p21 plays a part in autophagy regulation across different cell types. Al and colleagues [42] reported that p21 could influence the creation of LC3-II through direct interaction with LC3. Moreover, the antimalarial medication quinacrine promotes autophagy and apoptosis in breast cancer cells by modulating p21, which consequently inhibits tumor cell proliferation [43]. Hence, targeting p21 could be a means of indirectly regulating autophagy and affecting the anti-tumor ability of MPPα-PDT. Taken together, our findings indicate that PERK-signaling-dependent upregulation of p21 may counteract the pro-apoptotic effects of MPPα-PDT, whereas PERK signaling inhibition may enhance sensitivity to MPPα-PDT.

Meanwhile, there are some limitations to this study such as potential off-target effects or the necessity for further investigations to validate the observed effects, particularly in relation to the p21 pro-survival findings. Additionally, traditional PDT strategies have primarily focused on treating superficial cancers due to their non-invasive nature, high selectivity, and minimal side effects. The clinical application of PDT is currently limited to peripheral and endoscopically accessible areas (such as skin, neck, and mouth). However, the effectiveness of therapy for deep tumors is hindered by the limited tissue penetration depth of excitation light and poor *in vivo* targeting of photosensitizers.

**Figure 9:**
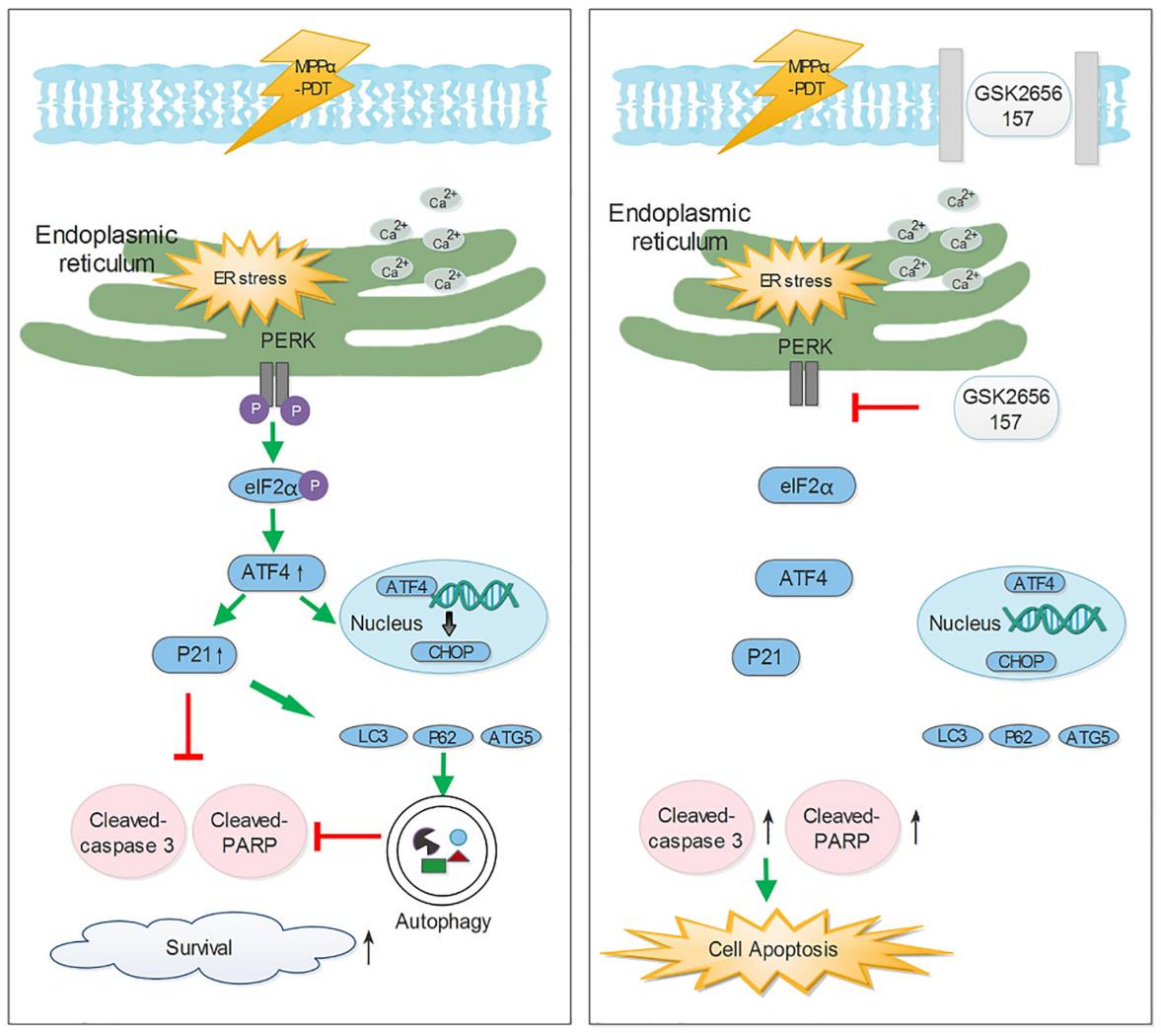
**The potential mechanisms of inhibiting Perk-ATF4-P21 pathway to enhance the efficacy of MPPα-PDT.** Antitumor ability of Mppα-PDT is improved by inhibiting PERK signaling, which is achieved by P21 regulating autophagy.

In conclusion (Figure 9), we sought to better understand how PERK signaling promotes the HOS cell survival induced by MPPα-PDT, as overcoming this may enhance sensitivity to MPPα-PDT. We found that MPPα-PDT combined with GSK2656157 enhances HOS cell apoptosis by suppressing autophagy. Crucially, this autophagy is p21-dependent, also indicating a pro-survival role of p21 during MPPα-PDT treatment. As inhibition of PERK signaling with GSK2656157 had similar results, it is possible that in the combined treatment group, GSK2656157 overcame p21-dependent autophagy to enhance HOS cells’ sensitivity to MPPα-PDT *in vivo* and *in vitro*.

## Supplementary Materials

Supplementary Figures
