# Supplementary material for: Targeting PERK-ATF4-P21 axis enhances the sensitivity of osteosarcoma HOS cells to Mppα-PDT
Source: Aging (Albany NY). 2024 Feb 5;16(3):2789–811. doi: 10.18632/aging.205511 (PMC10911341; doi:10.18632/aging.205511)
Supplement: Supplementary Figures [file aging-16-205511-s001.pdf]

SUPPLEMENTARY FIGURES

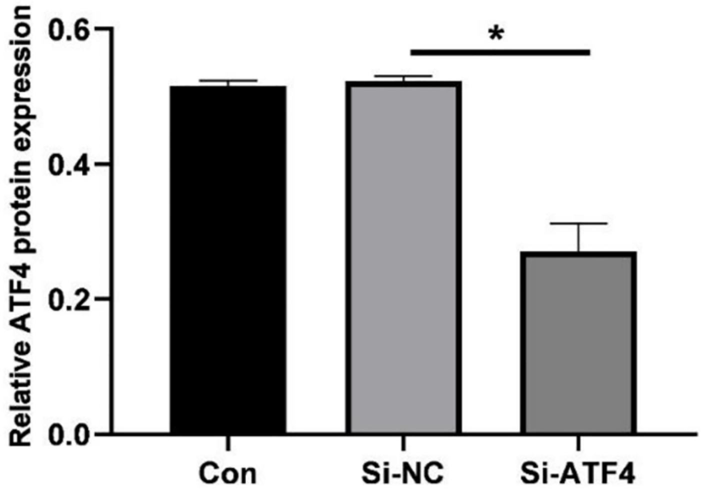

Supplementary Figure 1. Relative ATF4 protein expression.

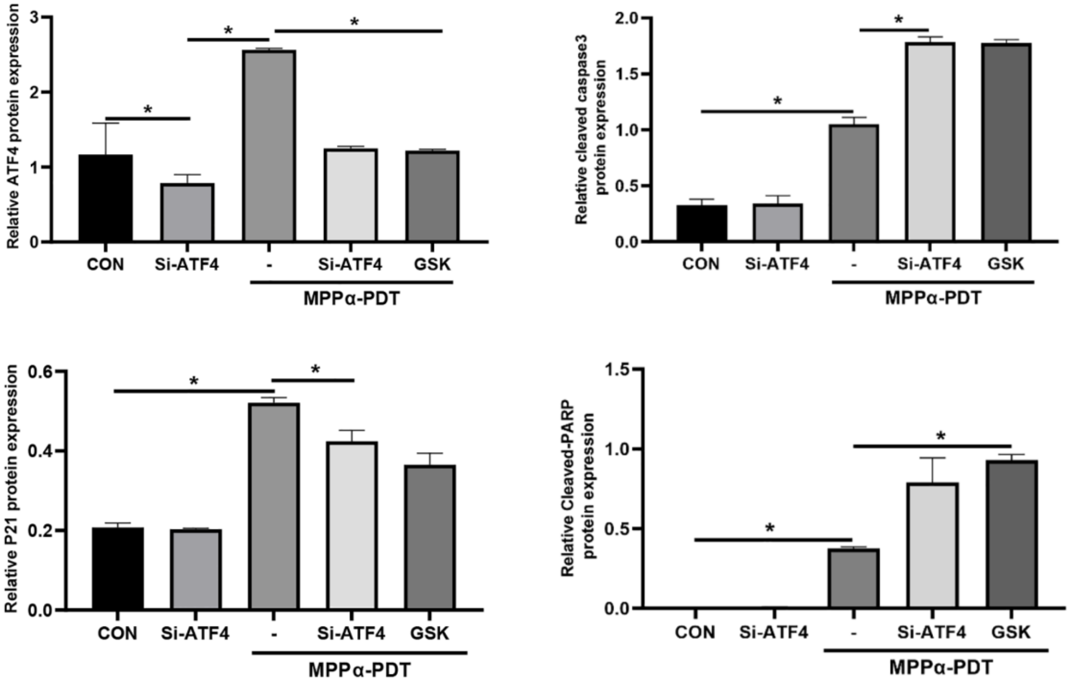

Supplementary Figure 2. Relative P21, cleaved-caspase3, ATF4, PARP proteins expression.

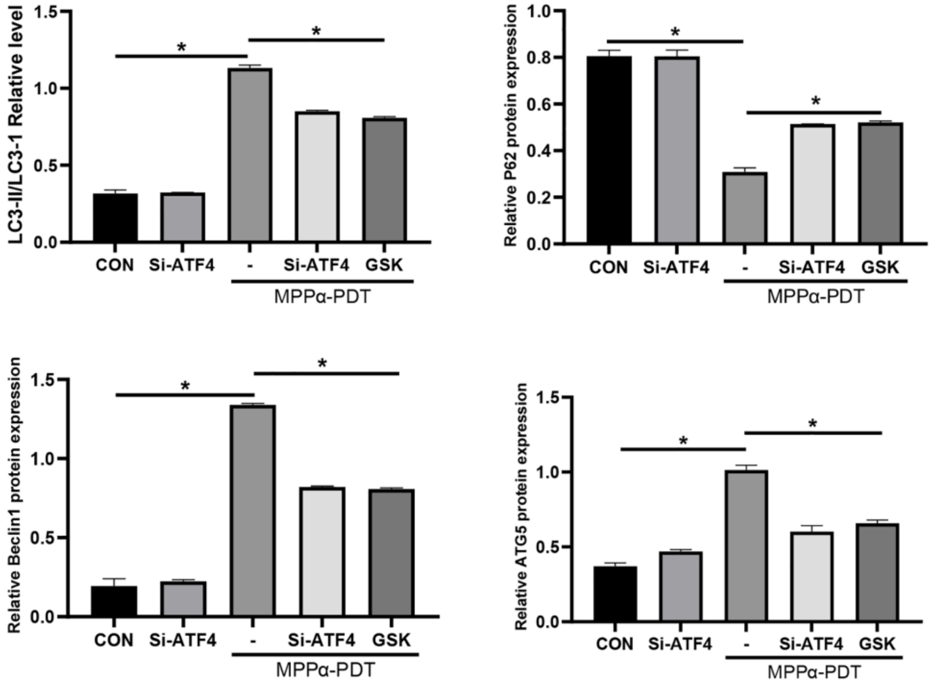

Supplementary Figure 3. Relative ATG5, P62, LC3II/LC3I, Beclin1 proteins expression.

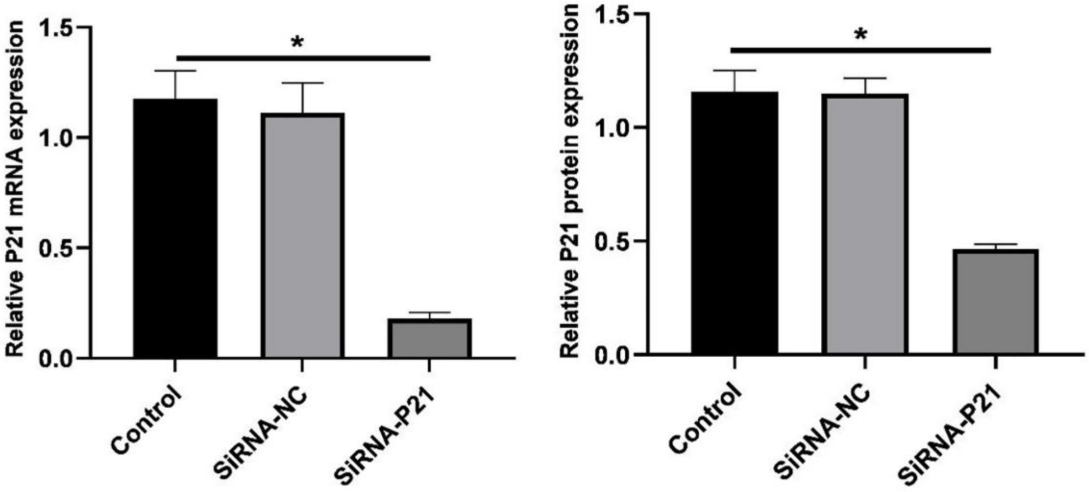

Supplementary Figure 4. Relative P21 protein expression and P21 mRNA expression.

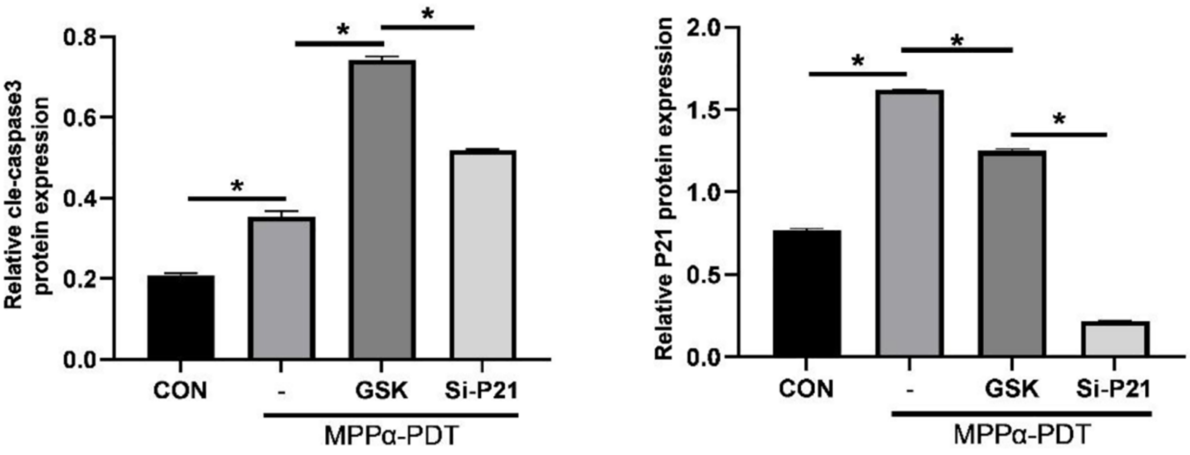

Supplementary Figure 5. Relative cleaved-caspase3 and p21 proteins expression.

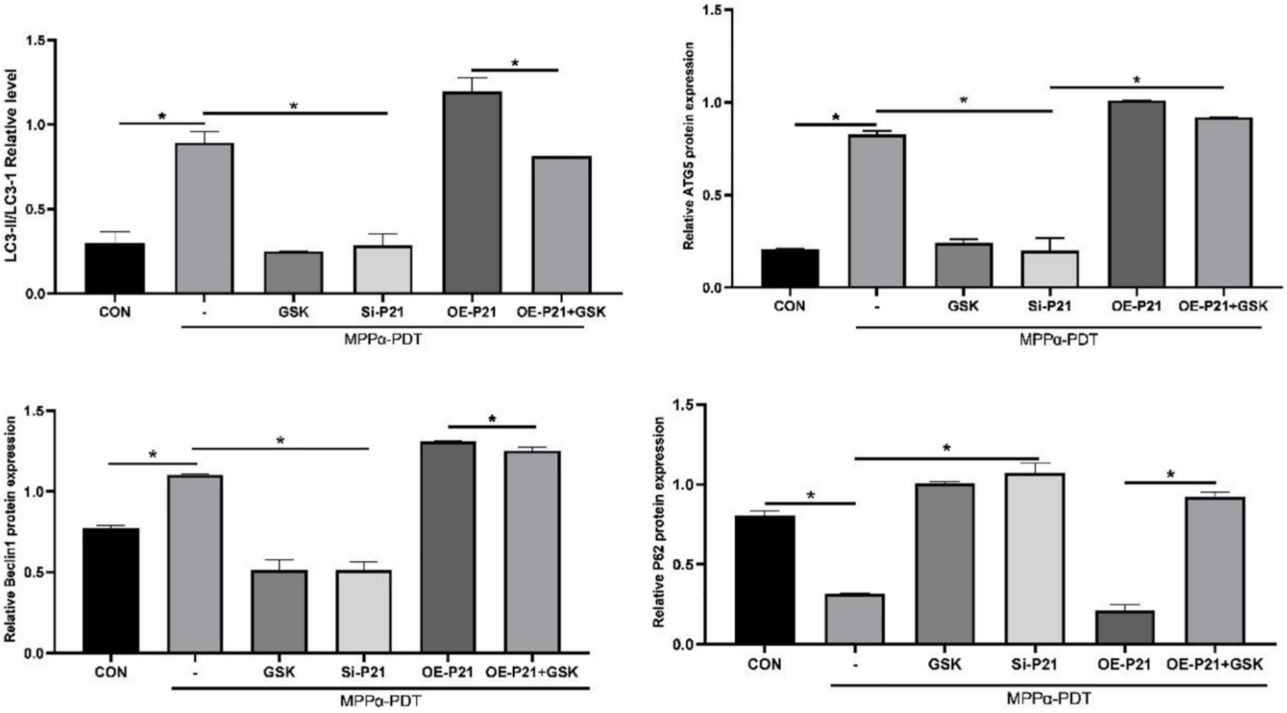

Supplementary Figure 6. Relative LC3II/LC3I, ATG5, Beclin1, P62 proteins expression.

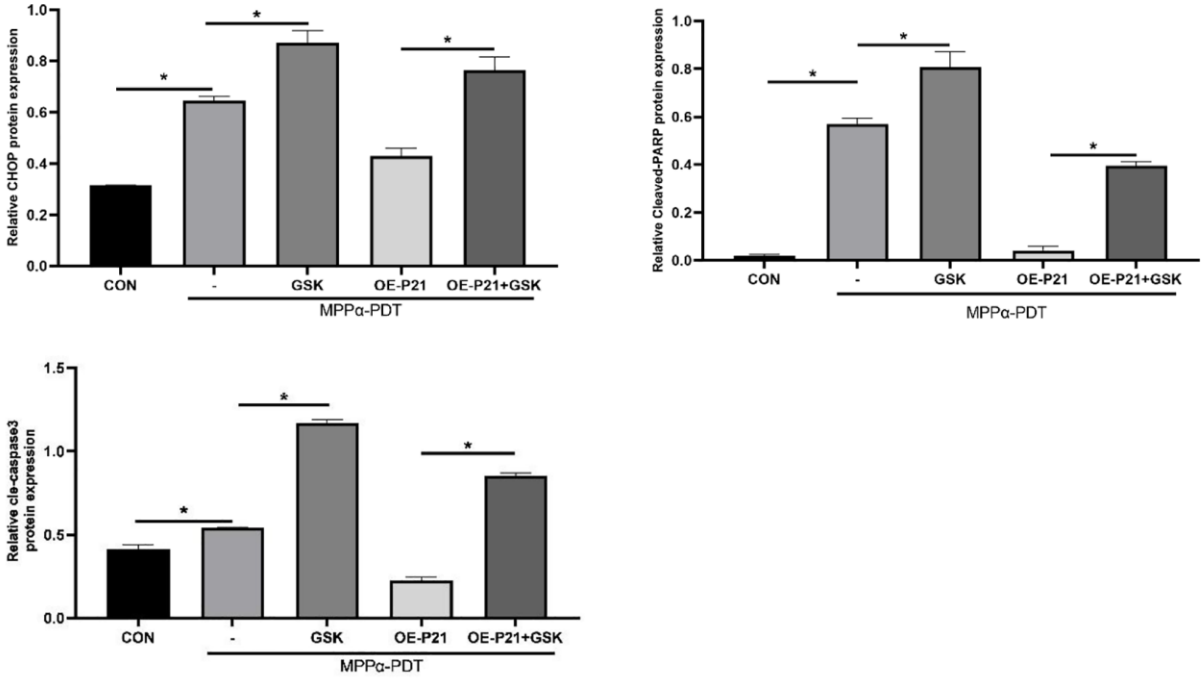

Supplementary Figure 7. Relative CHOP, cleaved-caspase3, cleaved-PARP proteins expression.
